# Management and outcome of adult generalized tetanus patients in a tertiary hospital in Anhui, China: a retrospective study

**DOI:** 10.3389/fpubh.2026.1735583

**Published:** 2026-04-17

**Authors:** Jizheng Huang, Erhui Cai, Wenying Ding, Cheng Dai, Hua Xiao, Wei Sun, Mingfeng Han

**Affiliations:** 1 Department of Hospital Infection Management, No.2 People’s Hospital of Fuyang City, Fuyang, China; 2 Clinical Research Center, The Second Affiliated Hospital of Shantou University Medical College, Shantou, Guangdong, China; 3 Department of Intensive Care Medicine, No.2 People’s Hospital of Fuyang City, Fuyang, China

**Keywords:** epidemiology, hospitalization costs, prognosis, tetanus, treatment

## Abstract

**Background:**

Tetanus is an acute, specific infectious disease associated with a high reported mortality rate and remains a major public health problem, particularly in low and middle-income countries. This study analyzes the epidemiological and clinical characteristics of adult tetanus patients admitted to our hospital to provide a basis for clinical treatment and prognosis.

**Methods:**

We conducted a retrospective analysis of the clinical data from 54 patients with tetanus admitted to a tertiary infectious disease hospital between January 1, 2018, and December 31, 2024. Disease severity was classified using the Ablett classification, which served as the primary grouping variable to categorize patients into the severe group and non-severe group. We analyzed the timing of patient presentation, cause of injury, trauma site, wound treatment prior to admission, disease classification, length of hospital stay, hospitalization costs, and mortality rate.

**Results:**

The mean age of 54 patients were 62.76 ± 12.69 years. The cohort included 30 male patients (55.56%) and 24 female patients (44.44%), with 51 patients (94.44%) from rural areas and 3 (5.56%) from urban areas. The mean hospital stay was 22.22 ± 13.38 days, the mean hospitalization cost was 67,297.44 ± 68,431.91 yuan, and 7 patients died (12.96%). A comparison of the treatment course between the non-severe and severe groups revealed statistically significant differences in several outcomes. The severe group had higher rates of ICU admission [0% (0/25) vs. 96.55% (28/29); *χ^2^* = 50.131, *p* < 0.001], mechanical ventilation [0% (0/25) vs. 89.66% (26/29); *χ^2^* = 46.552, *p* < 0.001], and pulmonary infection [28.00% (7/25) vs. 96.55% (28/29); *χ^2^* = 27.666, *p* < 0.001]. Furthermore, the severe group had a longer hospital stay [28.44 ± 14.82 days vs. 15.00 ± 6.16 days; *t* = −4.461, *p* < 0.001] and higher hospitalization costs (115,236.04 ± 60,894 yuan vs. 11,688.66 ± 4,145.69 yuan; *t* = −9.133, *p* < 0.001).

**Conclusion:**

Tetanus remains prevalent in rural areas and disproportionately affects older adults. Patients in the severe disease group experience substantial economic burden and higher mortality. Strengthening public awareness, improving wound care, and expanding immunization coverage, especially among rural older adults, are critical to reducing the disease burden.

## 1 Introduction

Tetanus is a severe infection caused by *Clostridium tetani*, a bacterium commonly found in the environment. The pathogen typically enters the body through wounds contaminated by soil, feces, or sputum. High-risk injuries include puncture wounds from nails or needles, burns, crush injuries, and wounds containing necrotic tissue. Less common routes of infection include superficial abrasions, surgical procedures, insect bites, dental infections, open fractures, chronic wounds, and intravenous drug use, among others (1, 2).

The incubation period for tetanus is typically short, ranging from 3 to 21 days, with most cases occurring within approximately 10 days. However, the period can vary from one day to several months, depending on the nature, extent, and location of the wound (3). Clinically, tetanus is characterized by muscle spasm. As the disease progresses, even minor stimuli can trigger generalized tonic convulsions, leading to complications and, in severe cases, death. The clinical forms of tetanus are primarily classified into three types: generalized tetanus, which accounts for approximately 88% of cases; localized tetanus, which accounts for about 12%; and cephalic tetanus, which is rare, accounting for roughly 1% (4). In generalized tetanus, symptoms typically begin with trismus (lockjaw) in the masticatory muscles, followed by involvement of the facial muscles, often resulting in a characteristic grimace known as risus sardonicus. The spasms then progress to the neck, back, abdominal, and limb muscles. Eventually, the diaphragm and intercostal muscles may be affected. Common manifestations include neck stiffness with the head tilted backward and involuntary contraction of the back muscles, causing the trunk to arch (opisthotonus). Involvement of the diaphragm can lead to cyanosis, respiratory distress, and potentially respiratory arrest (5, 6).

According to the Global Burden of Disease Study 2019, trauma-related tetanus was estimated to cause over 46,000 incident cases globally, resulting in approximately 28,000 fatalities. Most cases occurred in South Asia and sub-Saharan Africa (7). A systematic review of tetanus in Africa reported a crude case fatality rate as high as 43.2% (95% CI: 36.9%–49.5%) (8). Tetanus continues to pose a public health threat, a risk that can be exacerbated during natural disasters or urban flooding (4, 6). Therefore, summarizing the clinical epidemiology, diagnosis, treatment, and prevention of tetanus, as well as exploring strategies to reduce its incidence and mortality, remains a major public health priority.

Anhui Province, characterized by its agricultural landscape and large rural population engaged in farming, reports a relatively high incidence of trauma-related injuries, making tetanus an ongoing health concern. However, despite national surveillance data, detailed analyses of the clinical management, economic burden, and outcomes of adult tetanus—particularly when stratified by disease severity using the Ablett classification—in rural Chinese populations remained limited. In China, the current immunization program focuses primarily on childhood vaccination, and there is no routine booster policy for adults, especially among rural and older population. As a result, immunity often wanes with age, contributing to the observed demographic pattern of tetanus disproportionately affecting older adults in rural areas. This research aims to improve patient outcomes by enhancing diagnostic accuracy, refining treatment protocols, and strengthening prevention strategies, thereby helping to reduce both mortality and morbidity associated with the disease.

## 2 Materials and methods

### 2.1 Study design and patients

We retrospectively reviewed the electronic hospital database to identify adult patients with tetanus who were admitted to No.2 People’s Hospital of Fuyang City between January 1, 2018, and December 31, 2024. The diagnosis of tetanus was based on clinical assessment, in accordance with the World Health Organization (WHO) definition (9). This involved evaluating the patient’s wound history and confirming the presence of at least one of the following clinical signs: (i) lockjaw or risus sardonicus (a characteristic snarling smile), (ii) generalized or localized muscle spasms, or (iii) rigidity of the neck and/or abdominal muscles. Data on demographic information, clinical manifestations, vaccination status, and treatment were collected using a standardized case report form embedded in EpiData (version: 3.1, EpiData Association, Odense, Denmark).

### 2.2 Eligibility criteria

Inclusion criteria were: (1) age 18 years or older; (2) a history of a traumatic event or injury; (3) a clinical presentation of probable tetanus upon admission; and (4) a hospital stay exceeding 24 h.

Exclusion criteria were: (1) a subsequent ruling-out of the clinical diagnosis of tetanus, for instance, due to alternative diagnoses such as meningitis, rabies, or an oropharyngeal abscess; (2) pregnancy or lactation; (3) a comorbidity of autoimmune diseases, such as nephrotic syndrome or congenital immunodeficiency; (4) a diagnosis of a hematologic malignancy or ongoing chemotherapy; (5) incomplete medical records; or (6) patients who discharged within 24 h of hospital admission.

### 2.3 Disease severity classification

#### 2.3.1 Disease severity was assessed using the ablett grading system

Grade I: mild generalized spasms, mild-to-moderate trismus, no convulsions or respiratory distress; Grade II: moderate generalized spasms, moderate respiratory distress (RR > 30); Grade III: severe generalized spasms, severe dysphagia, continuous reflex spasms, HR > 120, severe respiratory distress (RR > 40); Grade IV: Grade III symptoms plus autonomic instability.

Severe cases were defined as Ablett III–IV; non-severe as I–II. The Ablett classification of all enrolled tetanus patients was independently assessed by two senior attending physicians (CD and WS) specializing in tetanus management. Discrepant cases were jointly reviewed and finalized by Chief Physician MH to minimize subjective bias and ensure grading consistency.

**Figure 1:**
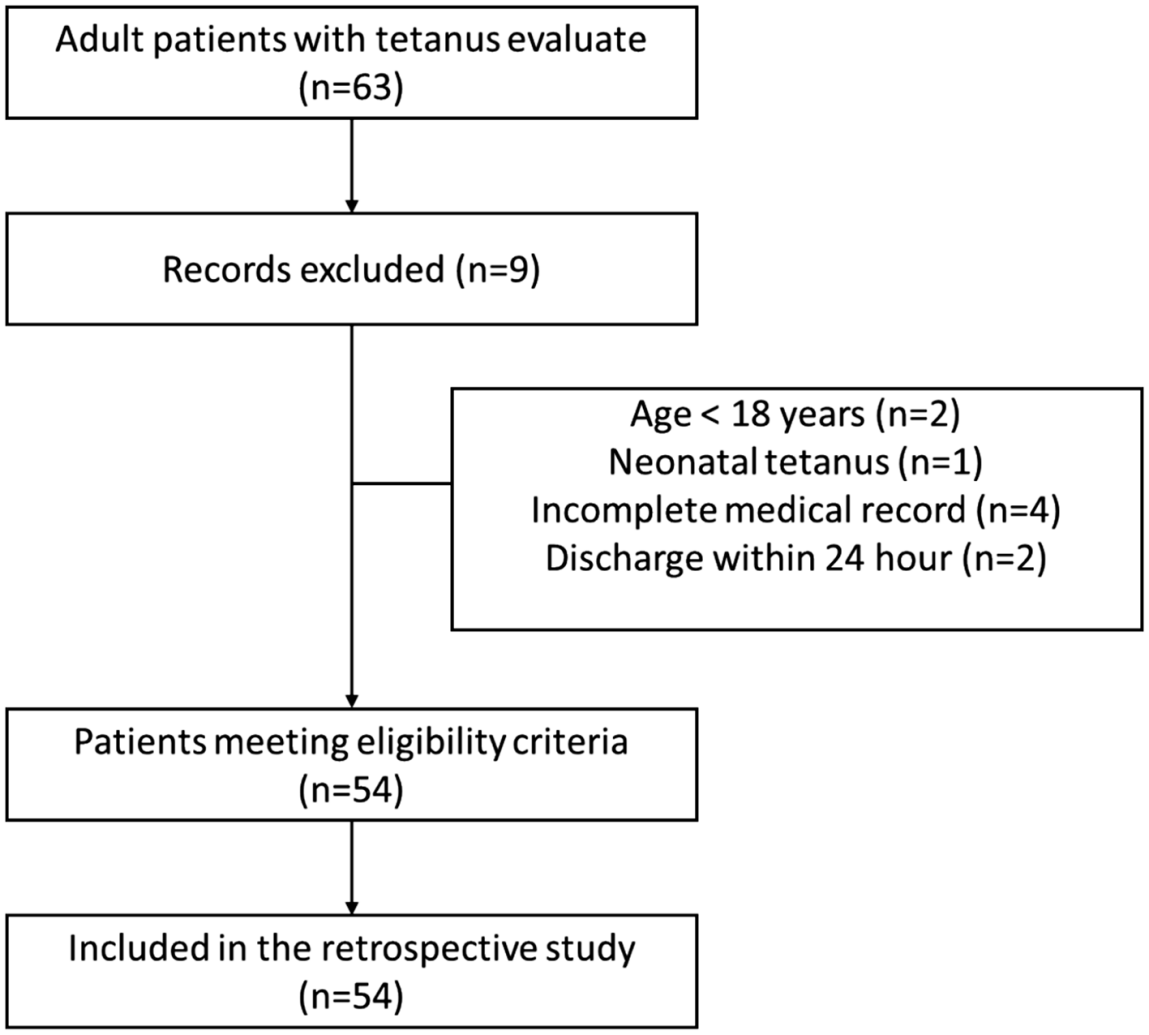
Flow chart of patient selection.

For analysis, all enrolled patients were categorized into two groups. The severe group comprised patients who were critically ill (Ablett grade 4) or had severe disease (Ablett grade 3), while the non-severe group included those with moderate (Ablett grade 2) and mild (Ablett grade 1) tetanus. The clinical outcomes assessed for the included tetanus cases were in-hospital mortality, disease deterioration, clinical improvement, and full recovery Figure 1.

### 2.4 Statistical analysis

Data analysis was performed using SPSS 25.0 statistical software (IBM Corporation, Chicago, IL, United States). A two-sided *p*-value of < 0.05 was considered statistically significant. For descriptive analysis, Continuous variables with a normal distribution were summarized as mean ± standard deviation (SD), those with a non-normal distribution were summarized as median and interquartile range (IQR), and categorical variables as frequencies and percentages. Between-group comparisons for normally distributed continuous variables were conducted using the independent samples t-test; non-normally distributed continuous variables were analyzed using the Mann–Whitney U test (Wilcoxon Rank-Sum test). Categorical variables were compared using the chi-square test or Fisher’s exact test, as appropriate. Multivariate logistic regression was performed to identify risk factors for severe tetanus, with odds ratios (OR) and 95% confidence intervals (CI) calculated. For clinical interpretability, SII was rescaled by dividing by 1,000, and OR were reported per 1,000-unit increase.

### 2.5 Ethic approval

This study was approved by the Ethics Committee of No.2 People’s Hospital of Fuyang City and was conducted in accordance with the principles of the Declaration of Helsinki. Due to the retrospective study design and the use of anonymized data, the ethics committee granted a waiver of written informed consent.

## 3 Results

### 3.1 Baseline demographics and clinical features

A total of 54 adult patients with tetanus were admitted to the hospital. Among these patients, the mean age was 62.76 ± 12.69 years (range: 41–91 years). The cohort included 30 male and 24 female patients, with a male-to-female ratio of 1.25:1. Most cases (51, 94.44%) were from rural areas, while only three (5.56%) were from urban areas. Vaccination history was obtained by inquiry from patients or family members at admission. Based on available medical records, no clear documentation of tetanus vaccination was found for any patient. The baseline demographic and clinical characteristics are summarized in Table 1.

**Table 1:** Demographic and baseline characteristics of included patients.

Variables	Non-severe group (*N* = 25)	Severe group (*N* = 29)	*p* value
Demographic features			
Mean age, years (±SD)	62.36 ± 13.90	63.10 ± 11.78	0.832
Sex			0.542
Male	15 (60.0%)	15 (51.7%)	
Female	10 (40.0%)	14 (48.3%)	
Area of residence			0.240*
Rural	25 (100.0%)	26 (89.7%)	
Urban	0 (0%)	3 (10.3%)	
Occupation			0.658*
Farmer	23 (92.0%)	25 (86.2%)	
Worker	2 (8.0%)	2 (6.9%)	
Other	0 (0%)	2 (6.9%)	
Disease timeline			
Incubation period, days (±SD)	9.88 ± 5.29	7.20 ± 2.10	0.028
Mean intervals from onset to admission, days (±SD)	4.24 ± 3.27	2.79 ± 1.82	0.057
Baseline clinical feature			0.507
Comorbidities	9 (36.0%)	8 (27.6%)	
Injury features			
Site of injury			0.852*
Foot	10 (40.0%)	13 (44.8%)	
Hand	9 (36.0%)	9 (31.0%)	
Oral cavity	1 (4.0%)	3 (10.3%)	
Leg	1 (4.0%)	2 (6.9%)	
Face	2 (8.0%)	1 (3.4%)	
Arm	2 (8.0%)	1 (3.4%)	
Cause of injury			0.254*
Injury by branches or crops	12 (48.0%)	6 (20.7%)	
Injury from metal objects	6 (24.0%)	5 (17.2%)	
Crush injury from heavy objects	1 (4.0%)	4 (13.8%)	
Fall from height	1 (4.0%)	3 (10.3%)	
Bicycle-related fall injury	0 (0%)	3 (10.3%)	
Electrical injury	0 (0%)	1 (3.4%)	
Dental extraction-related injury	1 (4.0%)	2 (6.9%)	
Postoperative infection	0 (0%)	1 (3.4%)	
Assault-related injury	1 (4.0%)	0 (0%)	
Unknown cause	3 (12.0%)	4 (13.8%)	
Wound management			0.120*
No-prehospital wound management	15 (60.0%)	18 (62.1%)	
Disinfection only	10 (40.0%)	7 (24.1%)	
Wound debridement and suturing	0 (0%)	4 (13.8%)	
Hospitalization burden			
Mean length of hospital stay, days (±SD)	15.00 ± 6.16	28.44 ± 14.82	< 0.001
Mean hospitalization cost, RMB (±SD)	11688.66 ± 4145.69	115236.04 ± 60894.83	< 0.001
Ablett classification at admission			0.001
I	16 (64.0%)	9 (31.0%)	
II	9 (36.0%)	6 (20.7%)	
III	0 (0%)	12 (41.4%)	
IV	0 (0%)	2 (6.9%)	

^*^Fisher’s exact test.

Data are presented as mean (SD), median (IQR), or *n* (%), unless otherwise indicated.

#### 3.1.1 Disease timeline

The incubation period ranged from 3 to 21 days, with a mean duration of 8.51 ± 4.18 days. The mean incubation period was shorter in the severe group (7.20 ± 2.10 days) than in the non-severe group (9.88 ± 5.29 days; *p* = 0.028). The interval from symptom onset to hospital admission ranged from 1 to 15 days, with an overall mean of 3.46 ± 2.67 days. Patients in the non-severe group presented to the hospital after a mean of 4.24 ± 3.27 days, compared to 2.79 ± 1.82 days for those in the severe group; however, this difference did not reach statistical significance (*p* = 0.057).

#### 3.1.2 Injury features

The majority of wounds involved extremities, with the foot being the most common site (23 cases, 42.59%), followed by the hand (18 cases, 33.33%). Other documented sites included the oral cavities (e.g., tooth extraction sites; 4 cases, 7.41%), leg and face (3 cases each, 5.56%), and arm (3 cases, 5.56%). The distribution of wound sites did not differ statistically significant between the two groups (*p* = 0.852).

#### 3.1.3 Causes of injury

Injury mechanisms were categorized as penetrating injuries, blunt trauma, procedure-related injuries, electrical injury, and unknown causes. Penetrating injuries were the most common, comprising 18 cases (33.33%) caused by branches or crops and 11 cases (20.37%) caused by metal objects. Blunt trauma included 5 cases (9.26%) due to heavy object impact and 4 cases (7.41%) due to falls, with three additional cases (5.56%) resulting from bicycle-related falls. Procedure-related injuries consisted of 3 cases (5.56%) following dental extractions and 1 case (1.85%) due to postoperative surgical site infection. One case (1.85%) of electrical injury was reported. The injury cause remained unknown in 7 cases (12.96%).

#### 3.1.4 Wound management

Prior to admission, only 4 patients (7.41%) had sought care at a local medical facility and undergone thorough debridement. Seventeen patients (31.48%) performed self-care with simple disinfection and dressing using agents such as alcohol or povidone-iodine, while the remaining 33 patients (61.11%) did not perform any wound management. None of the patients received tetanus antitoxin prophylaxis. No differences was observed in pre-hospital wound management between the non-severe and severe groups (*p* = 0.120).

#### 3.1.5 Disease severity and progression

On admission, 40 patients (74.1%) were classified as non-severe, and 14 (25.9%) as severe. Specifically, 25 cases (46.3%) were grade I, 15 cases (27.8%) as grade II, 12 cases (22.2%) as grade III, and 2 cases (3.7%) as grade IV. During hospitalization, disease progression occurred in 15 patients: 9 initially classified as grade I and 6 as grade II progressed to grades III or IV. All 15 patients with disease progression were transferred from the general ward to the intensive care unit (ICU), and 4 of them died. In total, 7 in-hospital death occurred in this cohort, resulting in a case fatality rate of 13.0%.

We defined a progression group (*n* = 15: mild/moderate at admission, later severe) and a non-progression group (*n* = 25: consistently mild/moderate). Compared with the non-progression group, the progression group had shorter incubation period, higher WBC, neutrophil, platelet, and SII levels (all *p* < 0.05) (Table 2).

**Table 2:** Comparison of indicators between clinical progression group and clinical non-progression group.

Variables	Clinical progression group (*N* = 15)	Clinical non-progression Group(*N* = 25)	Test statistic	*p* value
Mean age, years (±SD)	60.20 ± 12.35	62.36 ± 13.90	−0.496	0.623
incubation period, days (±SD)	6.86 ± 2.25	9.88 ± 5.29	−2.441	0.020
WBC^a^ (×10^9^∕L), median (IQR)	10.33 (7.49–13.37)	5.78 (4.95–7.61)	−4.037	<0.001
Neutrophil (×10^9^∕L), median (IQR)	7.60 (5.56–10.53)	3.80 (3.18–6.08)	−3.814	<0.001
Lymphocyte (×10^9^∕L), mean ± SD	1.64 (1.05–2.14)	1.50 (1.14–1.97)	−0.447	0.655
Platelets (×10^9^∕L), mean ± SD	287.00 (256.00–341.00)	214.00 (180.50–268.00)	−2.961	0.003
Serum albumin (×10^9^/L), mean ± SD	38.80 (35.70–42.90)	37.60 (34.85–39.35)	−0.992	0.321
Total Bilirubin (×10^9^∕L), median (IQR)	13.30 (11.60–20.80)	16.20 (10.30–19.25)	−0.279	0.780
Creatinine (×10^9^/L), median (IQR)	61.00 (54.00–67.00)	64.00 (52.50–69.50)	−0.504	0.615
Creatinine kinase (×10^9^/L), median (IQR)	297.00 (141.75–371.25)	172.00 (125.50–356.50)	−0.849	0.396
Creatine Kinase Isoenzyme (×10^9∕^L), median (IQR)	17.00 (16.00–27.50)	16.00 (12.00–27.00)	−0.909	0.363
SII^b^, median (IQR)	1336.17 (961.07–2720.01)	623.72 (415.25–825.84)	−3.311	0.001

^a^WBC, white blood cell.

^b^SII (Systemic immune-inflammation index) = Neutrophil count (×10^9^/L) × Platelet count (×10^9^/L)/Lymphocyte count (×10^9^/L).

#### 3.1.6 Hospitalization burden

Hospitalization burden was markedly greater in the severe group, as reflected by a longer mean hospital stay (28.44 ± 14.82 vs. 15.00 ± 6.16 days) and substantially higher mean hospitalization costs (RMB 115,236 ± 60,895 vs. RMB 11,689 ± 4,146) compared with the non-severe group (both *p* < 0.001).

#### 3.1.7 Laboratory parameters

A comparison of biochemical indices within 24 h of admission revealed that white blood cell count and neutrophil count were higher in the severe group than in the non-severe group (both *p* < 0.001). Notably, the systemic immune-inflammation index (SII) was higher in the severe group, with a mean of 2532.1 × 10^9^/L compared with 778.5 in the non-severe group (*t* = −3.907, *p* < 0.001), highlighting a substantial difference in systemic inflammatory response between the two groups.

Compared with the non-severe group, patients in the severe group had higher white blood cell counts and neutrophil counts (both *p* < 0.001). The inflammation index (SII) was also markedly elevated in the severe group (median 1559.74 vs. 623.72, *p* < 0.001). No statistically significant differences were observed between the two groups in lymphocyte count, platelet count, serum albumin, total bilirubin, creatinine, creatine kinase, or creatine kinase isoenzyme (all *p* > 0.05). Laboratory findings within 24 h of admission are summarized in Table 3.

**Table 3:** Comparison of laboratory parameters and systemic-inflammation index (SII) between severe and non-severe tetanus.

Variables	Non-severe group (*N* = 25)	Severe group (*N* = 29)	Test statistic	*p* value
WBC^a^ (×10^9^∕L), median (IQR)	5.78 (4.96–7.38)	10.13 (7.49–11.88)	−4.372	< 0.001
Neutrophil (×10^9^∕L), median (IQR)	3.80 (3.22–6.05)	8.86 (6.1–10.53)	−4.502	< 0.001
Lymphocyte (×10^9^∕L), mean ± SD	1.50 ± 0.57	1.28 ± 0.78	1.141	0.259
Platelets (×10^9^∕L), mean ± SD	221.04 ± 62.25	253.31 ± 86.88	−1.546	0.128
Serum albumin (×10^9^/L), mean ± SD	37.26 ± 4.19	39.56 ± 7.20	−1.455	0.152
Total Bilirubin (×10^9^∕L), median (IQR)	16.2 (10.4–18.9)	13.5 (11.7–20.2)	0.061	0.952
Creatinine (×10^9^/L), median (IQR)	64 (54.0–69.0)	61 (54.0–69.0)	0.365	0.715
Creatinine kinase (×10^9^/L), median (IQR)	172 (131.0–270.0)	313 (140.5–485.5)	−1.345	0.179
Creatine kinase lsoenzyme (×10^9∕^L), median (IQR)	16 (12.0–25.0)	19 (16.0–29.5)	−1.285	0.202
SII^b^, median (IQR)	623.72 (419.18–824.88)	1559.74 (1177.46–3174.74)	−4.276	< 0.001

^a^WBC: white blood cell.

^b^SII (Systemic immune-inflammation index) = Neutrophil count (×10^9^/L) × Platelet count (×10^9^/L)/Lymphocyte count (×10^9^/L.

#### 3.1.8 Multivariate logistic analysis of 54 tetanus patients

Multivariate logistic regression including age, sex, comorbidities, incubation period, injury site, wound management, and SII/1000 showed that per 1,000-unit increase in SII was an independent risk factor for severe tetanus (OR = 8.892, 95%CI:1.835–43.029, *p* = 0.007), while longer incubation period was a protective factor (OR = 0.680, 95%CI:0.471–0.981, *p* = 0.039) (Table 4).

**Table 4:** Multivariate logistic analysis of 54 tetanus patients.

Variables	B	S.E.	Wald	Sig.	Exp (B)	95% CI for EXP(B)	
Age	−0.004	0.040	0.011	0.916	0.996	0.921	1.076
Sex	−1.633	1.157	1.993	0.158	0.195	0.020	1.885
Comorbidities	−0.522	1.185	0.194	0.660	0.593	0.058	6.057
Incubation period	−0.386	0.187	4.253	0.039	0.680	0.471	0.981
Site of injury	−0.309	0.340	0.829	0.362	0.734	0.377	1.428
Wound management	0.887	0.872	1.035	0.309	2.428	0.440	13.413
SII/1000	2.185	0.805	7.365	0.007	8.892	1.835	43.092
Constant	4.205	4.258	0.975	0.323	67.042		

### 3.2 Treatment and management

Patients were managed using a comprehensive, multidisciplinary therapeutic approach that included wound debridement, antitoxin administration, antibiotic therapy, spasm control, management of autonomic dysfunction and hemodynamic instability with vasoactive drugs, airway support, nutritional and metabolic support, treatment of complications, and rehabilitation. The main treatment modalities and their frequencies are summarized in Table 5.

**Table 5:** Multidisciplinary clinical management in tetanus patients.

Treatment	Non-severe group (*N* = 25)	Severe group (*N* = 29)	*χ^2^* statistics	*p*
ICU admission			50.133	< 0.001
Yes	0 (0%)	28 (96.6%)		
No	25 (100%)	1 (3.4%)		
Mechanical ventilation			46.552	< 0.001
Yes	0 (0%)	26 (89.7%)		
No	25 (100%)	3 (10.3%)		
Vasoactive agents			—	< 0.001*
Yes	0 (0%)	11 (37.9%)		
No	25 (100%)	18 (62.1%)		
Pulmonary infection			27.666	< 0.001
Yes	7 (28.0%)	28 (96.6%)		
No	18 (72.0%)	1 (3.4%)		
Wound debridement			0.730	0.393
Yes	22 (88.0%)	23 (79.3%)		
No	3 (12.0%)	6 (20.7%)		
Antispasmodics			0.992	0.319
Yes	20 (80.0%)	26 (89.7%)		
No	5 (20.0%)	3 (10.3%)		
Analgesics and sedatives			—	< 0.001*
Yes	0 (0%)	27 (93.1%)		
No	25 (100%)	2 (6.9%)		
Neuromuscular blockades			—	< 0.001*
Yes	0 (0%)	15 (51.7%)		
No	25 (100%)	14 (48.3%)		
Tetanus antitoxin (TAT)			4.469	0.035
Yes	12 (48.0%)	22 (75.9%)		
No	13 (52.0%)	7 (24.1%)		
Tetanus immunoglobulin			2.444	0.118
Yes	13 (52.0%)	9 (31.0%)		
No	12 (48.0%)	20 (69.0%)		
Clinical outcome			—	0.012*
Favorable	25 (100%)	22 (75.9%)		
Unfavorable	0 (0%)	7 (24.1%)		

^*^Fisher exact test.

#### 3.2.1 ICU treatment

Among the 54 patients, 28 (51.85%) were treated in the ICU. None of the patients in the non-severe group required ICU admission, whereas 28 of 29 patients (96.6%) in the severe group were treated in ICU. This difference was statistically significant (*χ^2^* = 50.133, *p* < 0.001).

#### 3.2.2 Mechanical ventilation

Among the 54 patients, 26 (48.15%) received mechanical ventilation. No patients in the non-severe group required mechanical ventilation, compared with 26 patients (89.7%) in the severe group. This difference was statistically significant (*χ^2^* = 46.552, *p* < 0.001).

#### 3.2.3 Antibiotic use for pulmonary infection

Among the 54 patients, 35 (64.81%) developed pulmonary infections. Antibiotics for pulmonary infection were administered to 28 patients (96.55%) in the severe group compared with 7 patients (28.00%) in the non-severe group, indicating a substantially higher burden of secondary infection among severe cases. This difference was statistically significant (*χ^2^* = 27.666, *p* < 0.001).

#### 3.2.4 Wound management

All patients with an identifiable wound site underwent debridement to remove necrotic tissue and foreign material. Even wounds that appeared superficially healed were evaluated and repeated debridement was performed when necessary. Wounds were irrigated with hydrogen peroxide and normal saline and were managed either using an open technique or drainage, based on the clinical condition. Prompt and thorough debridement was considered essential to eliminate potential sources of infection. Debridement was typically omitted only when the wound was confirmed to be completely healed.

Among the 54 patients, 45 (83.33%) underwent wound debridement following admission. This procedure was performed in 22 patients (88.00%) in the non-severe group and 23 patients (79.31%) in the severe group. The difference in debridement rates between the two groups was not statistically significant (*χ^2^* = 0.730, *p* = 0.393).

#### 3.2.5 Neutralization of free toxin

Tetanus immune globulin (TIG) at a dose of 3,000–6,000 units was administered as a single intramuscular injection to neutralize unbound toxin when economically feasible. Alternatively, tetanus antitoxin (TAT) was administered intravenously at a dose of 30,000–50,000 units per day for 7–10 consecutive days.

Among the 54 patients, 34 (62.96%) received TAT and 22 (40.74%) received TIG. In the non-severe group, 12 patients (48.00%) were treated with TAT and 13 (52.00%) with TIG. In the severe group, 22 patients (75.86%) received TAT and 9 (31.03%) received TIG. The difference in TAT administration between groups was statistically significant (*χ^2^* = 4.469, *p* = 0.035), whereas the difference in TIG use was not statistically significant (*χ^2^* = 2.444, *p* = 0.118).

#### 3.2.6 Management of spasm, sedation, analgesia, and neuromuscular blockade

Patients with tetanus were maintained on strict bed rest in a quiet and calm environment, and external stimuli were minimized (e.g. noise reduction and use of blackout curtains) to prevent stimulus-induced muscle spasms. Benzodiazepines, such as diazepam and midazolam, were the main agents used for tetanic spasm control, and propofol was considered as in refractory cases.

Antispasmodics were administered in most patients in both groups. Specifically, 20 of 25 patients (80.0%) in the non-severe group and 26 of 29 patients (89.7%) in the severe group received antispasmodic therapy, with no statistically meaningful difference in the overall use between groups (*χ^2^* = 0.992, *p* = 0.319). However, the choice of antispasmodic therapy differed markedly by disease severity, with diazepam used predominantly in non-severe patients (20/25, 80%), whereas midazolam alone (20/29, 69.0%) or in combination with diazepam (7/29, 24.1%) was used almost exclusively in severe cases (only 2 used diazepam) (*χ^2^* = 46.69, *p* < 0.001).

Sedative use differed markedly between the two groups (*χ^2^* = 46.55, *p* < 0.001). None of the patients in the non-severe group received sedative therapy, whereas 27 of 29 patients (93.1%) in the severe group were sedated. Among sedated patients in the severe group, propofol was the predominant agent, used in 25 patients (86.2%), while remaining 2 patients (6.9%) received sedation without propofol.

Among patients requiring mechanical ventilation, analgesics were administered universally (26/29, 89.7%). Opioids, particularly remifentanil, were used as first-line agents to relieve pain and mitigate autonomic nervous system dysfunction. Analgesic use differed markedly between the two groups (*χ^2^* = 43.23, *p* < 0.001). None of the patients in the non-severe group received opioid analgesics, whereas 26 of 29 (89.7%) in the severe group were treated with opioids. Three severe patients (10.3%) did not receive any analgesic therapy.

Neuromuscular blocking agents were used exclusively in the severe group. In the severe group, 15 of 29 patients (51.7%) were treated with these agents. The difference in neuromuscular blocker use between the two groups was statistically significant (*p* < 0.001).

#### 3.2.7 Airway management

For patients whose muscle spasms were not adequately controlled with medication, early tracheotomy or endotracheal intubation served as a critical airway management strategy. Tracheotomy facilitated more effective airway suctioning, helped maintain adequate ventilation, and reduced the risk of pulmonary complications such as pneumonia.

Among the 54 patients in this study, 21 (38.89%) underwent tracheotomy after admission. No patients (0%) in the non-severe group required this procedure, compared with 21 patients (72.41%) in the severe group. This difference was statistically significant (*χ^2^* = 46.552, *p* < 0.001).

#### 3.2.8 Rational use of antibiotics

*Clostridium tetani* is a Gram-positive obligate anaerobic bacterium sensitive to penicillin and metronidazole. These antibiotics are commonly used, either alone or in combination, to reduce the release of tetanospasmin (tetanus toxin). In cases of secondary infection, antimicrobial therapy was adjusted based on culture results and susceptibility testing. In this study, all 54 patients (100%) received antibiotic therapy consisting of penicillin or amoxicillin combined with metronidazole.

#### 3.2.9 Nutritional and metabolic support

Reduced nutritional intake was commonly observed in patients with tetanus due to dysphagia, tracheotomy, or mechanical ventilation. Concurrently, the disease course was characterized by increased metabolic demands and substantial fluid loss from profuse sweating. These conditions predisposed patients to malnutrition, electrolyte disturbances, and acid–base imbalances. Supportive care, including adequate nutritional support and meticulous management of fluid, electrolyte, and acid–base balance, was therefore essential for improving outcomes and preventing complications.

### 3.3 Clinical and economic outcomes

No patients in the non-severe group required ICU admission. In contrast, 96.55% (28/29) of patients in the severe group were treated in the ICU. Among the 54 patients, 47 (87.04%) recovered and 7 (12.96%) died. All deaths occurred in the severe group. Patients with Ablett grade IV tetanus had a mortality rate of 44.44% Table 6.

**Table 6:** Prognosis and medical costs for inpatients with tetanus.

Ablett classification	ICU admission (%)	Mortality (%)	Length of stay (days)	Hospitalization costs (RMB)
I	0.00 (0/8)	0.00 (0/8)	10.25 ± 5.01	10288.00 ± 3957.53
II	0.00 (0/17)	0.00 (0/17)	17.24 ± 5.41	12347.80 ± 4181.56
III	95.00 (19/20)	15.00 (3/20)	29.25 ± 15.60	113145.39 ± 63657.24
IV	100.00 (9/9)	44.44 (4/9)	26.67 ± 13.62	119881.94 ± 57613.74
Total	51.85 (28/54)	12.96 (7/54)	22.22 ± 13.38	67297.44 ± 68431.91

## 4 Discussion

### 4.1 Demographics and public health implications

In this retrospective analysis of 54 adult tetanus patients from a tertiary hospital in Anhui Province, clear clinical and epidemiological distinctions were observed between severe and non-severe cases. Most patients were older adults from rural areas, and most injuries involved extremities, particularly the foot and hand, reflecting exposure to agricultural hazards. Despite the known effectiveness of vaccination, none of the patients had documented immunization or received tetanus prophylaxis prior to symptom onset, indicating substantial gaps in preventive care and public awareness.

Tetanus remained a preventable disease. In developed countries, comprehensive childhood vaccination programs and effective post-exposure prophylaxis have markedly reduced incidence (2, 10–12). However, in many low and middle-income regions, particularly in Asia and Africa, adult booster vaccination policies remain unclear or absent, contributing to a continued public health burden (13). Similar to our findings, multi-center studies from sub-Saharan Africa have reported high mortality among severe tetanus cases, with pooled case-fatality estimates exceeding 40% in hospitalized adults (8). While neonatal tetanus was eliminated in 2012, booster vaccination strategies for adolescents and adults have not yet been standardized. As a result, adults-especially older age groups—remain vulnerable.

The resulting clinical and financial burden remained substantial: In this cohort, the mean hospitalization cost reached RMB 67,297.44 with an average length of stay of 22.22 days, with an overall case fatality rate of 12.96%. Patients with severe disease incurred a considerably higher mean treatment cost of RMB 115,236.04 and required prolonged hospitalization duration (mean: 28.44 days); those who required mechanical ventilation needed it for an average of approximately 20.67 days. The economic and clinical burden were concentrated among the severe cases, consistent with the previous report from China and elsewhere (14–16).

### 4.2 Clinical severity and outcomes

Disease severity was strongly associated with multiple clinical indicators: patients in the severe group demonstrated substantially shorter incubation periods, markedly higher systemic immune-inflammation index (SII), and greater need for intensive interventions, including ICU admission, mechanical ventilation, sedation, and analgesia (17). Likewise, a recent multicentered analysis from Bangladesh identified shorter incubation time, respiratory compromise, and the need for mechanical ventilation as major predictors of mortality, closely aligning with our observation that severe cases required intensive airway management and had notably higher fatality rates (18). Importantly, all deaths occurred in the severe group, underscoring the prognostic significance of early severity stratification using the Ablett classification. Together, these findings highlight the ongoing burden of tetanus in resource-limited rural settings and emphasize the need for improved prevention strategies, earlier recognition of severe disease, and optimized supportive care.

Despite older age, occupational and geographic factors further contribute to vulnerability. Most participants were farmers living in rural areas (92.60%), and many injuries received inadequate wound management. Only 4 patients (7.41%) underwent proper debridement prior to admission, and none received passive immunization with tetanus immunoglobulin. These findings suggested missed opportunities for primary and secondary prevention (18).

Notably, 15 of the 54 patients (27.8%) initially presenting with mild or moderate tetanus subsequently progressed to severe disease—a finding with profound clinical implications. This substantial rate of deterioration underscores the unpredictable and potentially fulminant nature of tetanus, even in patients who appear stable at admission. There remains a need for objective markers to support early severity prediction (17). The Ablett classification, while widely used, is qualitative and dependent on clinician assessment. The Systemic Immune-Inflammation Index (SII) reflects systemic inflammation, prothrombotic state, and impaired adaptive immunity, with prognostic value in critical infections (19). Pathophysiologically, tetanospasmin induces neutrophilia, lymphopenia, and thrombocytosis, which directly increase SII (1, 17). Our research also suggests that SII is a potential inflammatory biomarker. In the future, larger cohorts need to be studied to verify as an auxiliary and complementary biomarker.

### 4.3 Clinical implications for treatment and resources use

Tetanus continues to carry a high fatality rate. Without medical intervention, mortality among severe cases approaches 100%, even with aggressive treatment, reported mortality rate still ranges from 10% to 50% (18, 20, 21). In this study, 29 patients were classified with severe tetanus. Except for one patient whose family declined intensive care, all severe cases received ICU treatment, and 21 (72.41%) underwent tracheotomy. The case fatality rates for Ablett grade III and IV were 15.00 and 44.44%, respectively. The overall case fatality rate in this cohort was 12.96%, aligns with reports from Slovenia (12.9%) (22) but exceeds those from Suzhou, Jiangsu (6.3%), Fujian Province (5.23%) (14, 15, 23), and Guangzhou (0%). These differences may reflect variations in the timeliness of medical intervention, socioeconomic factors, and availability of critical care resources.

Our economic analysis reveals a striking disparity in treatment costs: the mean hospital expenditure for a severe tetanus case in this cohort was RMB 115,236, which is more than tenfold higher than that for non-severe cases (RMB 9,842). This order-of-magnitude cost difference highlights the substantial clinical and financial burden of severe tetanus on patients and healthcare systems, especially in resource-limited rural areas where most patients resided. Given that 27.8% of initially mild or moderate cases progressed to severe disease, even clinically stable patients face considerable downstream economic risks. Proactive vaccination could effectively reduce avoidable morbidity, mortality, and economic costs.

## 5 Limitations

This study has several limitations. First, its retrospective design may introduce certain information biases. Second, as a single-center study with a relatively small sample size, the findings may be influenced by regional variations in diagnostic and treatment practices. Therefore, the research results should be interpreted as associative rather than causal.

## 6 Conclusion

Post-traumatic tetanus remains a serious health threat in rural areas and continues to impose a substantial clinical and economic burden, particularly in severe and critical cases. In China, the lack of active immunization strategies for adolescents and adults, combined with limited awareness of post-exposure passive immunization, contributes to preventable morbidity and mortality. To address this gap, public health efforts should focus on implementing catch-up tetanus vaccination for adults—especially individuals over 50 years of age in rural communities—and integrating tetanus prophylaxis education into primary care systems and agricultural worker safety programs.

## Data Availability

The original contributions presented in the study are included in the article/supplementary material, further inquiries can be directed to the corresponding authors.
